# Root pruning improves maize water-use efficiency by root water absorption

**DOI:** 10.3389/fpls.2022.1023088

**Published:** 2023-01-04

**Authors:** Minfei Yan, Cong Zhang, Hongbing Li, Li Zhang, Yuanyuan Ren, Yinglong Chen, Huanjie Cai, Suiqi Zhang

**Affiliations:** ^1^ State Key Laboratory of Soil Erosion and Dryland Farming on the Loess Plateau, Northwest Agriculture and Forestry University, Yangling, Shaanxi, China; ^2^ College of Forestry, Northwest Agriculture and Forestry University, Yangling, Shaanxi, China; ^3^ School of Pharmacy, Weifang Medical University, Weifang, China; ^4^ Geography and Environmental Engineering Department, Baoji University of Arts and Sciences, Baoji, China; ^5^ The University of Western Australia Institute of Agriculture, and University of Western Australia School of Agriculture and Environment, The University of Western Australia, Perth, WA, Australia; ^6^ Key Laboratory of Agricultural Soil and Water Engineering in Arid and Semiarid Areas, Ministry of Education, Northwest Agriculture and Forestry University, Yangling, China

**Keywords:** abscisic acid, jasmonic acid, leaf water potential, root hydraulic conductivity, root pruning

## Abstract

Root systems are an important component of plants that impact crop water-use efficiency (WUE) and yield. This study examined the effects of root pruning on maize yield, WUE, and water uptake under pot and hydroponic conditions. The pot experiment showed that root pruning significantly decreased root/shoot ratio. Both small root pruning (cut off about 1/5 of the root system, RP1) and large root pruning (cut off about 1/3 of the root system, RP2) improved WUE and root hydraulic conductivity (Lpr) in the residual root system. Compared with that in the un-cut control, at the jointing stage, RP1 and RP2 increased Lpr by 43.9% and 31.5% under well-watered conditions and 27.4% and 19.8% under drought stress, respectively. RP1 increased grain yield by 12.9% compared with that in the control under well-watered conditions, whereas both pruning treatments did not exhibit a significant effect on yield under drought stress. The hydroponic experiment demonstrated that root pruning did not reduce leaf water potential but increased residual root hydraulic conductivity by 26.2% at 48 h after root pruning under well-watered conditions. The foregoing responses may be explained by the upregulation of plasma membrane intrinsic protein gene and increases in abscisic acid and jasmonic acid in roots. Increased auxin and salicylic acid contributed to the compensated lateral root growth. In conclusion, root pruning improved WUE in maize by root water uptake.

## 1 Introduction

Roots are the principal organs responsible for water and nutrient uptake in plants. They help maintain water homeostasis in the entire plant, responding to changing external environments (Hirte et al., 2018). Root systems can adjust to the ambient environment and help the whole plant adapt to different conditions. The improvement of root function is necessary to increase water uptake by the plant (Meister et al., 2014). The root system is an important index to determine crop water-use efficiency (WUE) (Sreeman et al., 2018; Corales et al., 2020), which is vital for sustaining productivity and minimising crop water utilisation.

Passioura (1983) proposed that crop yield and WUE do not necessarily increase with the size of the root system. Until now, the relationship between root size and yield and WUE of crops has been controversial. Some studies advocated that larger root systems can absorb more water, resulting in higher yield and WUE (Ehdaie et al., 2010; Palta et al., 2011). In contrast, other studies indicated that root systems with a small biomass and root length were unintentionally selected in modern cultivars aiming for higher yield (Song et al., 2009; Aziz et al., 2017; Fang et al., 2021).

Root pruning is the artificial reduction of root biomass by excising parts of the root system. Research on root pruning has mainly focused on relationships between the timing and extent of root pruning and their effects on crop yield and above-ground plant growth. However, no consistent patterns have been observed or reported. Decreasing root/shoot ratio (R/S) by root pruning improved grain yield and WUE significantly (Ma et al., 2010; Hu et al., 2019). Wang et al. (2007) reported that root pruning could increase grain yield under drought, but not under sufficient water supply. Additionally, vertical root pruning decreased grain number and grain weight (Xu et al., 2016). Plant responses to root pruning are complex and encompass many aspects resulting in changes in growth and biomass allocation patterns. Root pruning has been reported to promote the growth of fine roots (Feng et al., 2022) and flowering (Budiarto et al., 2019). Further, root pruning can reduce nutrient and water competition between roots and grains (Fanello et al., 2020). Photosynthetic traits were also improved significantly by root pruning (Liu et al., 2007). The responses to root pruning may differ among species and/or when additional environmental factors interact with root pruning. Drought is considered to be the primary limiting factor among these interacting factors. However, previous studies have not evaluated the hydraulic properties of plants with pruned roots; thus, the responses in maize-to-root water uptake function after root pruning under different water conditions remain unclear.

Root hydraulic conductivity (Lpr) reflects the ability of roots to absorb water from the soil (Knipfer et al., 2011; Sánchez-Romera et al., 2018). The presence of aquaporins (AQPs) in cell membranes plays an important role in the regulation of Lpr (Meng and Fricke, 2017). The plasma membrane intrinsic protein (PIP) aquaporin subfamily includes the PIP1 and PIP2 subfamilies and regulates root water uptake (Lee et al., 2012; Perrone et al., 2012). When PIP was downregulated in *Arabidopsis thaliana* or *Nicotiana tabacum*, root water absorption was partially reduced and slowly recovered after rehydration (Martre et al., 2002). Therefore, PIPs regulate hydraulic conductance and resistance. Nevertheless, the specific functions of certain PIPs and their synergistic effects require further investigation.

Plant hormones are believed to be involved in the regulation of Lpr and water uptake (Aroca, 2006). The contents of abscisic acid (ABA) and jasmonic acid (JA) in roots were significantly increased by root pruning (Feng et al., 2022). In roots, ABA enhanced Lpr and improved plant water status (Tardieu et al., 2010; Olaetxea et al., 2015). Additionally, ABA may participate in long-distance signal transduction between roots and shoots and directly affect water conductivity between these organs and tissues (Aroca et al., 2003). ABA might also control plant water status by regulating root hydraulic conductance and transpiration rate and by inducing genes governing intracellular dehydration tolerance (Zhang et al., 2006; Yao et al., 2019). JA is a critical signalling molecule involved in plant growth, development, and stress responses (Qi et al., 2015; Du et al., 2017). It might affect water conductivity and interact with ABA to mediate these processes (Adie et al., 2007; Barrero et al., 2009). Phytohormones control root growth and maintain overall plant water balance. Therefore, it is important to examine the relationships between root growth and phytohormone content after root pruning. This information can help determine the mechanisms by which root growth function changes in response to root pruning.

In the present study, we investigated the effects of root pruning on yield, WUE, and water absorption capacity of maize under different water conditions. We conducted a soil-filled pot experiment and polyethylene glycol (PEG)-induced drought experiment in solution to determine water absorption. We hypothesised that (1) root pruning can improve yield and WUE of maize, (2) root pruning can enhance hydraulic conductivity of the residual root, and (3) the increase in root hydraulic conductivity is associated with aquaporin activity and changes in phytohormone levels.

## 2 Materials and methods

### 2.1 Plant materials and experimental design

#### 2.1.1 Pot experiment

A pot experiment was conducted between May and September 2019 in Yangling, China. Maize (*Zea mays* L.) var. Qinlong 14 was used in both the pot and hydroponic experiments. Seeds were disinfected with 2% (w/v) sodium hypochlorite, rinsed with sterile distilled water, and placed in a germination chamber in the dark at 28°C. After three days, the germinated seeds were planted in a plastic pot (height, 27 cm; diameter, 28 cm). Each pot was filled with 17 kg sieved topsoil and a polyvinyl chloride (PVC) tube (diameter, 1.5 cm) for irrigation. Loamy clay soil was collected from the top 0–20 cm of cropland. The soil organic carbon content, total nitrogen, available phosphorus, available potassium, pH, and bulk density were 13.1 g kg^− 1^, 0.78 g kg^− 1^, 13.8 mg kg^− 1^, 124.6 mg kg^− 1^, 7.4, and 1.37 g cm^− 3^, respectively. All pots were supplied with 200 mg kg^−1^ CH_4_N_2_O and 150 mg kg^−1^ KH_2_PO_4_. Two seeds were sown in each pot and thinned to one seedling per pot seven days after sowing. There were 16 replicates per treatment.

**Figure 1:**
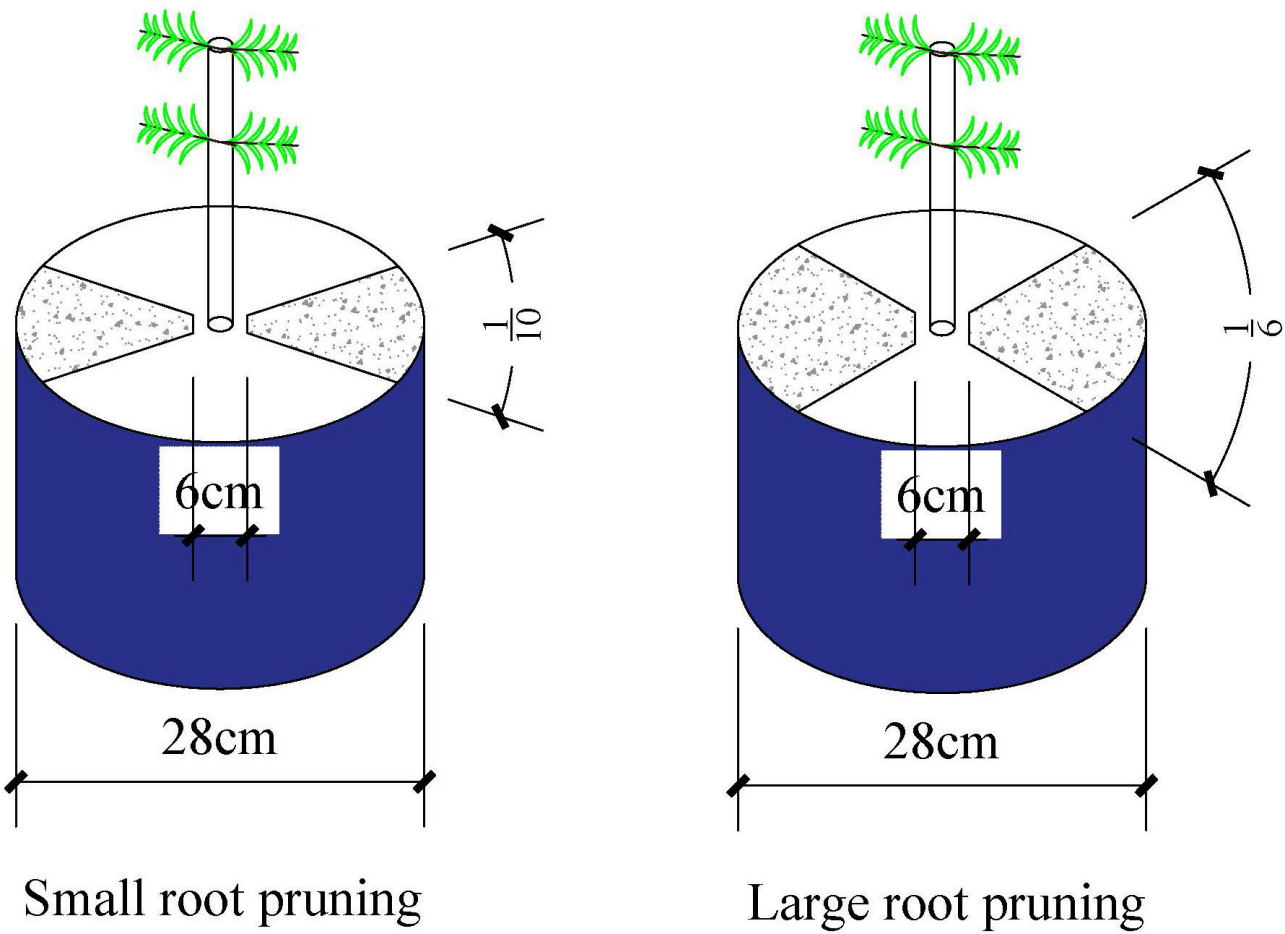
The root pruning method.

At the maize five-leaf stage (24 days after sowing), the plants were either well-watered (WW; 75–85% field water capacity) or subjected to drought stress (DS; 35–45% field water capacity). Drought stress was induced by stopped the supply of water, and the relative soil water content was maintained by water to weight at 6:00 pm daily. At the maize six-leaf stage (30 days after sowing), the plants were subjected to the following root pruning treatments: (1) small root pruning (RP1) (cut off about 1/5 root system); (2)large root pruning (RP2) (cut off about 1/3 root system); and (3) no root pruning (R0) (control). The root system was cut off from the soil vertically from the soil surface to the bottom along the along the shaded edges of the **Figure 1** (the assigned percentage shaded portion is shown in **Figure 1**) and approximately 3 cm away from the plant using a 28 cm single-sided knife (the soil was not removed from the pot). The assigned percentage areas for RP1 and RP2 are illustrated in **Figure 1**. Plant samples were collected at the jointing stage (V6, 32 days after sowing), anthesis (V12, 58 days after sowing), milk stage (R3, 83 days after sowing) and maturity (R6, 107 days after sowing).

#### 2.1.2 Hydroponic experiments

The hydroponic experiment was conducted in an artificial climate chamber (AGC-D001P; Qiushi Corp., Beijing, China). Seeds of maize var. Qinlong 14 were disinfected with 2% (w/v) sodium hypochlorite, rinsed with sterile distilled water, and placed in a germination chamber at 28°C in the dark for three days. Uniform germinated seeds were selected and transplanted into the hydroponic in a plastic container (38 cm × 28 cm × 15 cm) filled with ½ Hoagland nutrient solution (pH 6.0). Six plants were transplanted into the six holes (2.5 cm in diameter) on a foam board (37 cm × 27 cm × 1.5 cm) in each container. Plants were grown in a glasshouse with mean day/night temperatures of 25 and 20°C and a relative humidity of 65 and 55%, respectively, with a daylight photon flux density of 400 μmol m^-2^ s^-1^ for 13 h (06:30–19:30).

The 1/3 root pruning treatment was chosen to explore the mechanism of root pruning on root hydraulic conductivity following our initial trials involving various root cutting proportions (**Figure S1**). PEG6000 was used to simulate drought stress (Haswell and Verslues, 2015; Hellal et al., 2018). The following treatments were applied 10 days after sowing: (1) well-watered conditions (WW-R0); (2) PEG6000 (10% W/V) (PEG-R0), the osmotic potentials of the nutrient solutions were -0.30 Mpa and measured using a dew point penetrator (Model 5520, Wescor, Logan, UT, USA); (3) removal of approximately 1/3 root system from the base (WW-RP); (4) removal of approximately 1/3 root system from the base and exposure to PEG6000 (10%) (PEG-RP). The nutrient solution was refreshed every three days and aerated for 13 h per day by an air pump. After root pruning, each incision was immediately sealed with paraffin. Plant samples were collected at 3, 6, 12, 24, and 48 h after treatment. The experiments were repeated three times, and each repetition comprised at least three biological replicates.

### 2.2 Measurements

#### 2.2.1 Root hydraulic conductivity measurement

For the hydroponics experiment, Lpr was measured using a pressure chamber (Type 3005, Soil Moisture Equipment Corporation, Santa Barbara, CA, USA). The roots of six maize seedlings were excised 2 cm above the middle hypocotyl and fixed in the sample chamber. The steel plug was tightened, and the pressure was increased from 0 to 0.5 MPa in 0.1 MPa increments. The xylem sap discharged every 0.1 MPa was absorbed with cotton wool. There was 1 min equilibration between adjacent pressure levels. The volume of the xylem sap was determined based on its mass, and a scanner (Epson Perfection V800, Seiko Epson Crop., Suwa, Japan) was used to calculate root surface area. The root water absorption capacity at each pressure step was expressed as the water yield per unit root surface area per unit time. Samples were collected at 3, 6, 12, 24, and 48 after treatment, and six replicates were measured.

In the pot experiment, Lpr was determined using the high-pressure flow metre (HPFM-Gen3, Dynamax Inc., Houston, TX, USA). Seedlings were excised at the first internodes, the pressure coupler was connected to the cutting site, and the air was evacuated. The pressure was increased to ~300 MPa at a rate of 2–5 MPa s^-1^. The relationship between flow velocity pressure and time was measured. Roots were selected, rinsed, and scanned, and their surface areas were determined. Plant samples were collected at the jointing, anthesis, and milk stages, and six replicates were measured.

Lpr was calculated as follows:


(1) $$Lpr=V\times S^{-1}\times P^{-1}\times t^{-1}$$

where Lpr is the root hydraulic conductivity (m s^-1^ MPa^-1^), V is the total volume of water passing through the root (m^3^), S is the root surface area (m^2^), P is the external pressure (MPa), and t is time (s).

The pre-dawn water potential of the newer fully expanded leaf was measured in a pressure chamber (Type 3005; Soil Moisture Equipment, Santa Barbara, CA, USA).

#### 2.2.2 Water-use efficiency

WUE in pot experiments was calculated based on the following equation:


(2) WUE (g/Kg)=GY/ET

where GY is the grain yield per pot at maturity; ET is transpiration rate and the recorded total water consumption per pot over the whole growing cycle.

#### 2.2.3 *ZmPIPs* expression level

The expression levels of the aquaporin genes *ZmPIP*1:1, *ZmPIP*1:2, *ZmPIP*1:3, *ZmPIP*1:4, *ZmPIP*1:5, *ZmPIP*2:1, *ZmPIP*2:2, *ZmPIP*2:4, *ZmPIP*2:5, and *ZmPIP*2:6 in the hydroponics experiment were measured by RT-qPCR. Root samples were collected 2-10 cm away from the root tip at 3 h, 6 h, 12 h, 24 h, and 48 h after treatment, immersed in liquid nitrogen, and stored at d stored at −80°C. Total RNA was extracted with TRIzol reagent. The cDNA was synthesised with a SuperRT cDNA Synthesis Kit (Kangwei Biology Co., Ltd., Jiangsu, China). Each sample was reacted at 37°C for 90 min and 85°C for 5 s according to the manufacturer’s instructions. Each sample was then added to the UltraSYBR mixture (Kangwei Biology Co., Ltd.) according to the manufacturer’s instructions. The PCR was run in a 7300 Real-Time PCR instrument (Applied Biosystems, Foster City, CA, USA) using the following programme: rapid activation at 95°C for 10 min; 40 cycles at 95°C for 15 s; and 60°C for 1 min. The dissolution curve was set at 95°C for 15 s, 60°C for 1 min, 95°C for 15 s, and 60°C for 15 s. The internal reference gene was *GADPH*, and a standard curve was plotted to determine the relative changes in gene expression. There are three biological replicates.

#### 2.2.4 Abscisic acid, jasmonic acid, indole-3-acetic acid and salicylic acid extraction, purification, and quantification

For the hydroponics experiment, approximately 0.5 g fresh roots were powdered, mixed with 4 mL pre-cooled 80% (v/v) methanol containing 200 mg L^-1^ di-*tert*-butyl-*p*-methylphenol and 500 mg L^-1^ citric acid monohydrate, and shaken at 4°C in the dark overnight. The suspensions were centrifuged at 10,000 × *g* and 4°C for 15 min, and the supernatants were collected. Then, 3 mL pre-cooled 80% (v/v) methanol was added to each precipitate again, and each suspension was shaken at 4°C in the dark for 2 h. The suspensions were centrifuged at 10,000 × *g* and 4°C for 15 min, and the supernatants were collected. The supernatants were combined and lyophilised in a freeze-dryer (FreeZone Plus 2.5, Labconco Corp., Kansas, MO, USA) and dissolved in 600 µL 80% (v/v) methanol. The phytohormone levels were determined by high-resolution ion-mobility liquid mass spectrometry (LC-30A+TripleTOF5600+; AB SCIEX, Singapore). The samples were injected into a BEH C18 column (1.8 µm; 2.1 mm × 100 mm). The mobile phases were (a) 0.05% (v/v) acetic acid and (b) 0.05% (v/v) acetic acid in acetonitrile. Three biological replicates were analysed per treatment.

#### 2.2.5 Root sampling and measurements

The roots were picked, rinsed, and scanned using a scanner (Epson Perfection V800, Seiko Epson Crop.) with a transparency adapter at 300 dpi. Root surface area, root length, and root volume were analysed using analysis software (WinRHIZO, Regent Instrument Inc., Québec, QC, Canada). All root samples were oven-dried at 75°C for 48 h and weighed on an analytical balance.

Root absorption area was determined using the methylene blue adsorption method. Optical density of the adsorbed solution was read at 660 nm, and the root absorption area was determined by interpolation against a standard curve.

#### 2.2.6 ABA and JA inhibitor treatments

The JA inhibitor sodium diethyldithiocarbamate trihydrate (DIECA, 100 mM) and the ABA inhibitor fluridone (10 mM) were added to the nutrient solutions. The DIECA and fluridone crystals were first dissolved in 5 ml of 0.5% (v/v) ethanol and then added to a 6 L nutrient solution. The final ethanol concentration in the nutrient solution was 0.00041% (v/v). Control plants were grown in the nutrient solution with the same amount of ethanol [0.00041% (v/v)] (Luo et al., 2019). Roots and leaves were sampled for each treatment after 3, 6, 12, 24, and 48 h of treatment.

### 2.3 Statistical analysis

SPSS v. 14.0 (SPSS Inc., Chicago, IL, USA) was used for data processing. Treatment means were compared by Duncan’s multiple range tests. Differences between means were considered significant at *P*< 0.05. SigmaPlot v. 12.5 (Systat Software Inc., Chicago, IL, USA) was used for graph plotting and analysis of correlations between indices.

## 3 Results

### 3.1 Root biomass and root/shoot ratio

The proportion of pruned roots was determined after pruning (**Table 1**). The root biomass and the R/S significantly decreased in response to root pruning under different moisture conditions. In the pot experiments, approximately 1/5 of the roots were subjected to small pruning root (RP1) and approximately 1/3 of the roots were subjected to large pruning root (RP2). In the hydroponics experiment, approximately 1/3 of the roots were subjected to pruning root (RP). Hence, the actual proportions of pruned roots approximately met expectations.

**Table 1:** Root dry weight (R), root/shoot ratio (R:S), and proportion of pruned root (RP).

	Treatments		Root(g/plant)	R:S	RP(%)
Pot experiment	WW	R0	26.38a	0.45a	
		RP1	21.46b	0.38b	18.66% ± 2.24%
		RP2	19.02c	0.33c	28.34% ± 2.61%
	WS	R0	24.91a	0.47a	
		RP1	20.33b	0.39b	18.16% ± 1.68%
		RP2	17.91c	0.34c	29.01% ± 2.04%
Hydroponics experiment	WW	R0	0.24a	0.20a	
		RP	0.16b	0.13b	35.20% ± 3.82%
	PEG	R0	0.23a	0.23a	
		RP	0.15b	0.15b	36.56% ± 2.38%

For the pot experiment, R0 is no root pruning, RP1 is small root pruning, RP2 is large root pruning and WW means well-watered conditions and DS means drought stress. For the hydroponics experiment, R0 is no root pruning, RP is root pruning and WW means well-watered conditions and PEG means PEG stress. Measurements were made two days after root pruning (jointing stage) in the pot experiment and 48 h after root pruning in the hydroponic experiment. Data are means of five replicates. Different letters in the same column indicate significant difference at P< 0.05 according to Duncan’s test.

### 3.2 Yield and water use efficiency

**Table 2** shows that under well-watered conditions, the yield and 100-grain weight of RP1increased by 12.9% and 6.4%, respectively. Root pruning did not exhibit a significant effect on yield or ear length under drought stress. Under drought stress, however, root pruning improved maize 100-grain weight by 9.3% (RP1) and 19.1% (RP2).

**Table 2:** Effects of root pruning on yield, ear length, 100-grainweight, transpiration rate in response to irrigation throughout the growth period (ET), and water use efficiency (WUE) for grain yield.

Treatments		Yield (g pot^-1^)	Ear length (cm)	100-grainweight (g)	ET (Kg)	WUE (g/Kg)
WW	R0	148.01b	15.58a	36.51b	48.62a	3.07bc
	RP1	167.06a	16.62a	38.83a	45.01b	3.71a
	RP2	143.88b	15.07a	36.75b	43.77b	3.35b
DS	R0	85.9c	9.5b	29.12d	26.44c	3.25bc
	RP1	89.52c	10.66b	31.83c	23.37d	3.83a
	RP2	83.77c	10.87b	34.68b	22.81d	3.68a
Probability level of ANOVE						
W		**	**	*	**	**
P		**	NS	*	**	*
W × P		*	NS	*	NS	NS

For the pot experiment, R0 is no root pruning, RP1 is small root pruning, and RP2 is large root pruning. WW indicates well-watered and DS indicates drought stress. Data are means (n = 6). Different letters indicate significant differences among treatments (P< 0.05) based on Duncan’s test. ANOVA results for the main factors (water, W; root pruning, P) and their interactions (W × P) are given for each parameter.*, P<0.05; **, P< 0.01; NS, no significant.

Root pruning reduced water consumption under different soil moisture levels throughout the maize growth period; transpiration rates of RP1 and RP2 decreased by 7.4% and 10.0%, respectively, under well-watered, and 11.6% and 13.7%, respectively, under drought stress (**Table 2**).

Compared with the unpruned control plants, the pruned plants exhibited greater WUE under both soil moisture levels. WUE of RP1 and RP2 increased by 20.8% and 9.1%, respectively, under well-watered, and 17.8% and 13.2%, respectively, under drought stress (**Table 2**).

Grain yield, ear length, 100-grain weight, transpiration rates, and WUE were significantly affected by water treatment (*P*< 0.05); grain yield, 100-grain weight, transpiration rates, and WUE were significantly affected by root pruning treatment (*P*< 0.05). There was no significant water pruning interaction for ear length, transpiration rates, and WUE (**Table 2**).

### 3.3 Root hydraulic conductivity

**Figure 2:**
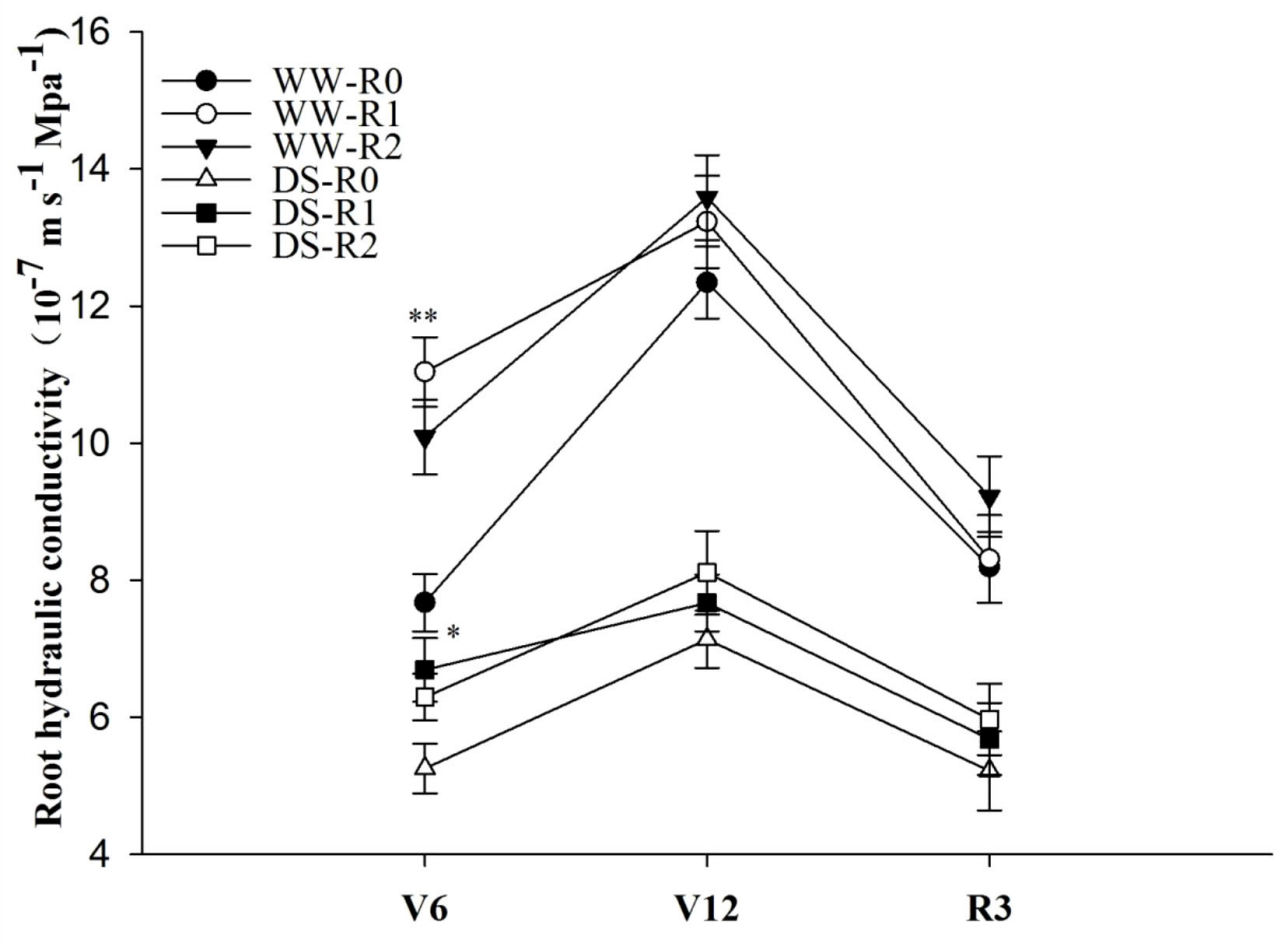
Effects of root pruning on root hydraulic conductivity (Lpr) in the pot experiment. WW-R0 is no root pruning under well-watered conditions; WW-RP1 is small root pruning under well-watered conditions; WW-RP2 is large root pruning under well-watered conditions; DS-R0 is no root pruning under drought stress; DS-RP1 is small root pruning under drought stress; DS-RP2 is large root pruning under drought stress. Samples were measured at jointing stage (V6), anthesis (V12), and milk stage (R3). Values are means ± standard error (n=6). The asterisks indicate significant differences by independent *t*-tests under the same moisture conditions (* *P<* 0.05; ** *P<* 0.01).

In the pot experiment, root pruning significantly (*P*< 0.01) enhanced the Lpr of maize at the jointing stage. Compared to that of the control, at the jointing stage, the Lpr of RP1 and RP2 were 43.9% and 31.5% higher under well-watered conditions and 27.4% and 19.8% higher under drought stress, respectively (*P*< 0.05) (**Figure 2**). Drought stress significantly (*P*< 0.01) inhibited Lpr, exhibiting a 31.5% reduction compared to that in the well-watered non-pruned treatment. The Lpr of pruned plants was not significantly (*P* > 0.05) different from that of the control at the anthesis and milk stages.

**Figure 3:**
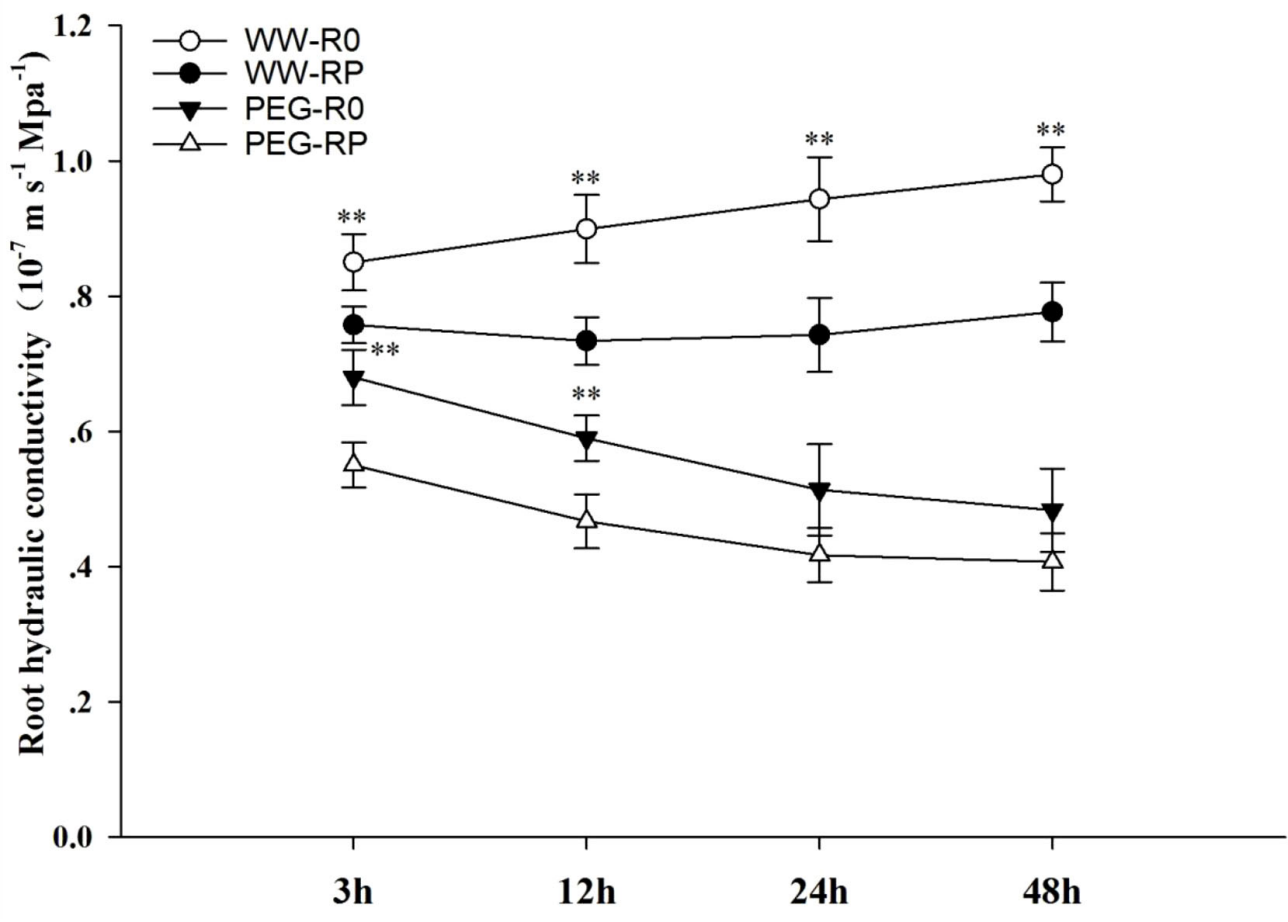
Effects of root pruning on Lpr in the hydroponic experiment. WW-R0 is no root pruning under well-watered conditions; WW-RP is removal of 1/3 of the root system under well-watered conditions; PEG-R0 is no root pruning under PEG stress; PEG-RP is PEG stress plus removal of 1/3 of the root system at 3 h, 12 h, 24 h, and 48 h after treatment. Values are means ± standard error (n = 6). The asterisks indicate significant differences by independent *t*-tests under the same moisture conditions (** *P<* 0.01).

In the hydroponic experiments, root pruning significantly (*P*< 0.01) increased maize Lpr by 12.2%, 22.6%, 27.0%, and 26.2% at 3, 12, 24, and 48 h after root pruning under the well-watered condition, respectively (**Figure 3**). Lpr was significantly (*P*< 0.01) lower under PEG stress than under the well-watered condition. Under PEG stress, Lpr was significantly lower in pruned plants than in the control at early hours after PEG treatment (*P*< 0.05).

### 3.4 Leaf water potential

**Figure 4:**
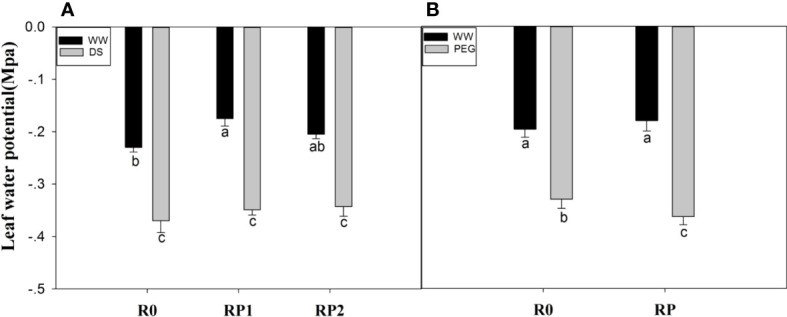
Effects of root pruning on leaf water potential in the pot experiment **(A)** and in the hydroponic experiment **(B)**. Measurements were made at jointing stage in the pot experiment and 48 h after root pruning in the hydroponic experiment. Data for five biological replicates were analysed by ANOVA. Different letters indicate significant differences from each other (*P*< 0.05). The treatment abbreviations are defined in **Figures 2**, **3**.

In the pot experiment, under well-watered conditions, the leaf water potential was 23.9% (RP1) and 10.87% (RP2) higher than that control; however, no such difference was observed under drought stress (**Figure 4A**). In the hydroponic experiments, PEG stress reduced leaf water potential of pruned plants by 10.1% than PEG-R0 (**Figure 4B**).

### 3.5 Root absorption areas and active absorption areas

**Figure 5:**
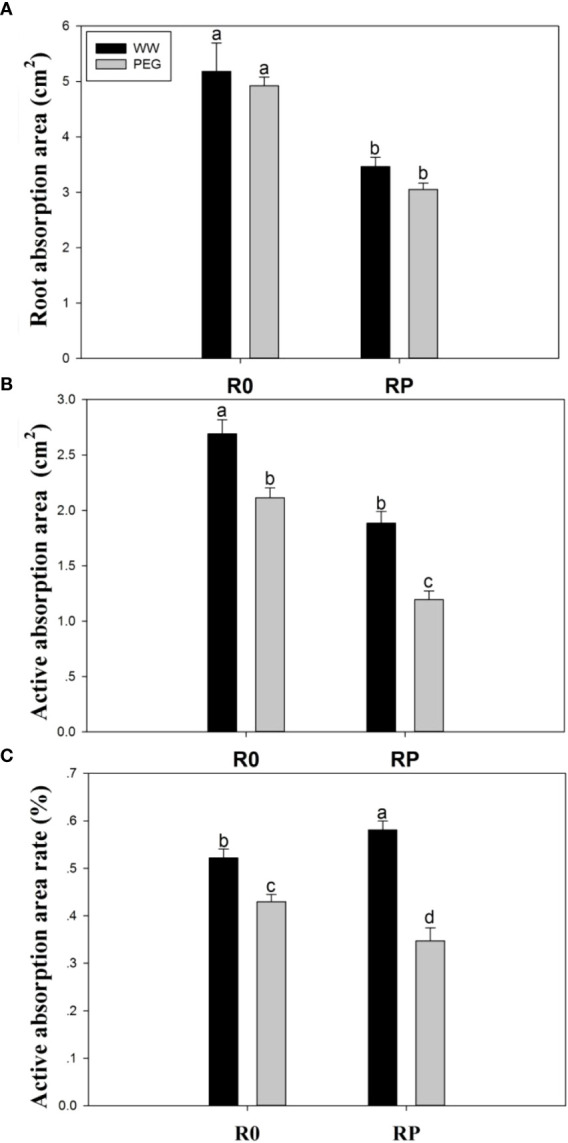
Effects of root pruning on root absorption surface **(A)**, root active absorption area **(B)**, and root active absorption area ratio **(C)** in the hydroponic experiment. Data for five biological replicates were analysed by ANOVA. Different letters indicate significant differences from each other (*P*< 0.05). The treatment abbreviations are defined in **Figure 3**.

The absorption area (**Figure 5A**) and the active absorption area (**Figure 5B**) significantly decreased after root pruning, because this treatment reduced the root biomass. The active absorption area was reduced by 21.5% (R0) and 55.6% (RP) under PEG stress compared with that of R0 under well-watered conditions, respectively (**Figure 5B**). However, the active absorption area ratio under WW-RP was 11.3% higher than under WW-R0 (**Figure 5C**).

### 3.6 Root *ZmPIP* expression

**Figure 6:**
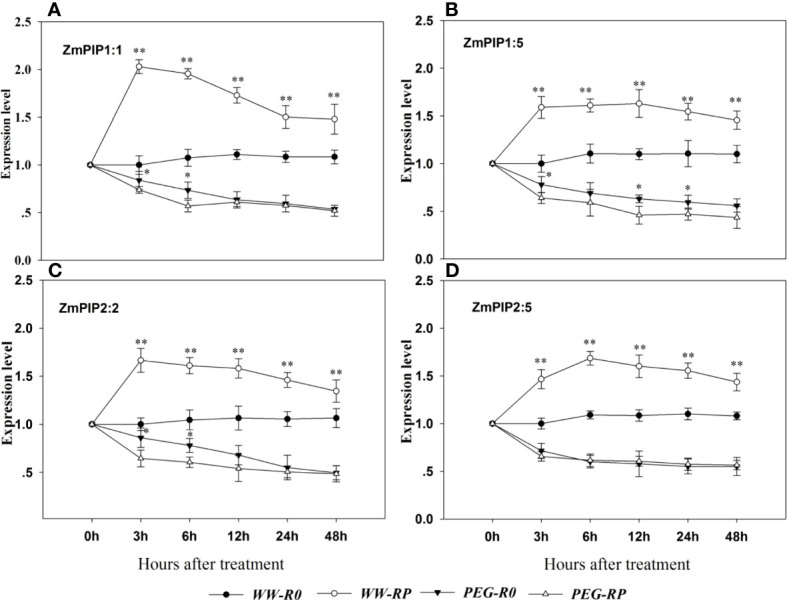
Effects of root pruning on *ZmPIP* expression. *ZmPIP*1:1 **(A)**, *ZmPIP1*:5 **(B)**, *ZmPIP*2:2 **(C)**, and *ZmPIP*2:5 **(D)** measured by RT-qPCR at 3, 6, 12, 24 and 48 h after root pruning. Data for three biological replicates were analysed by ANOVA. The treatment abbreviations are defined in **Figure 3**. The asterisks indicate significant differences by independent *t*-tests under the same moisture conditions (* *P<* 0.05; ** *P<* 0.01).

The expression levels of five *ZmPIP*1 and five *ZmPIP*2 in the root system were measured at 0, 3, 6, 12, 24, and 48 h after treatment (**Figure 6**). Among them, *ZmPIP*1:1, *ZmPIP*1:5, *ZmPIP*2:2 and *ZmPIP*2:5 were the most abundant genes. Under well-watered conditions, the expression level significantly increased after root pruning and peaked at 3 h. The expression levels of *ZmPIP*1:1, *ZmPIP*1:5, *ZmPIP*2:2 and *ZmPIP*2:5 were 2.0-fold, 1.6-fold, 1.7-fold, and 1.5-fold higher, respectively, in the pruned plants (WW-RP) than in the unpruned plants (WW-R0) at 3 h.

Most of the foregoing genes were downregulated after the PEG treatment (PEG-R0). The expression levels of *ZmPIP*1:1, *ZmPIP*1:5, *ZmPIP*2:2 and *ZmPI*P2:5 in R0 at 3 h after PEG treatment were 16.0%, 22.0%, 14.0%, and 28.5% lower than in R0 under well-watered conditions, respectively. *ZmPIP*1:1, *ZmPIP*1:5, *ZmPIP*2:2, and *ZmPIP*2:5 were downregulated in pruned plants at 3 h after PEG treatment. However, the differences in *ZmPIP* expression between R0 and RP gradually reduced with treatment beyond 3 h, and at 48 h, there were no significant differences in the expression levels of *ZmPIP*1:1 or *ZmPIP*2:5 between pruned and non-pruned plants after 12 h under PEG stress.

### 3.7 Root phytohormone content

**Figure 7:**
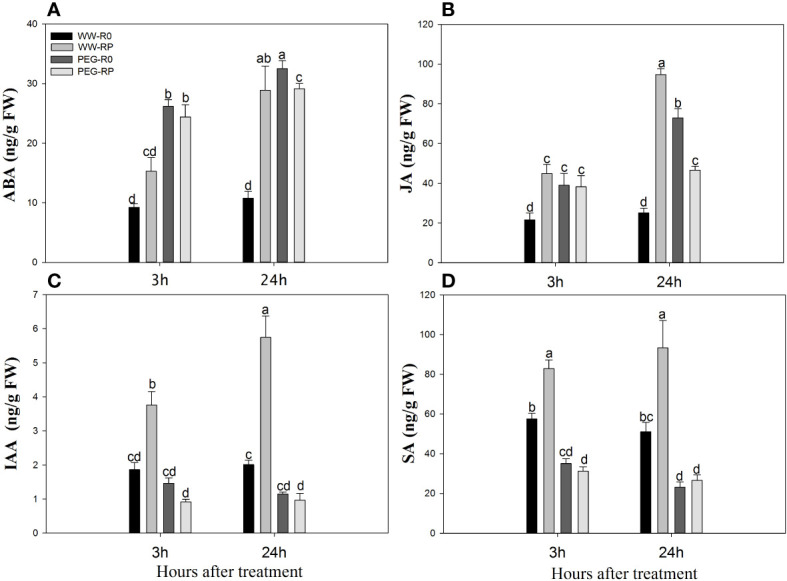
Levels of abscisic acid (ABA) **(A)**, jasmonic acid, (JA) **(B)**, auxin (IAA) **(C)** and salicylic acid (SA) **(D)** in roots changed at 3 h and 24 h after root pruning in the hydroponic experiment. Data for three biological replicates were analysed by ANOVA. Different letters indicate statistically significant differences from each other (*P*< 0.05). FW, fresh weight. The treatment abbreviations are defined in **Figure 3**.

Root ABA and JA levels of WW-RP were 1.7–2.7-fold and 2.1–3.8-fold higher than those of WW-R0 between 3 h and 24 h, respectively (**Figures 7A, B**), and root IAA and SA levels of WW-RP were 2.0–2.9-fold and 1.4–1.8-fold higher than those of WW-R0, respectively (**Figures 7C, D**).

PEG stress significantly increased the relative root ABA and JA content. Root ABA and JA levels in PEG-R0 were 2.8–3.0-fold and 1.8–2.9-fold higher than those in WW-R0 between 3 h and 24 h, respectively; and root ABA and JA levels in PEG-RP were 2.6–2.7-fold and 1.8–1.9-fold higher than those in WW-R0, respectively (**Figures 7A, B**). PEG stress lowered the relative root IAA and SA levels whereas root pruning under PEG stress (PEG-RP) did not induce any elevation (**Figures 7C, D**).

### 3.8 Effects of inhibitors on root water conductivity after pruning

**Figure 8:**
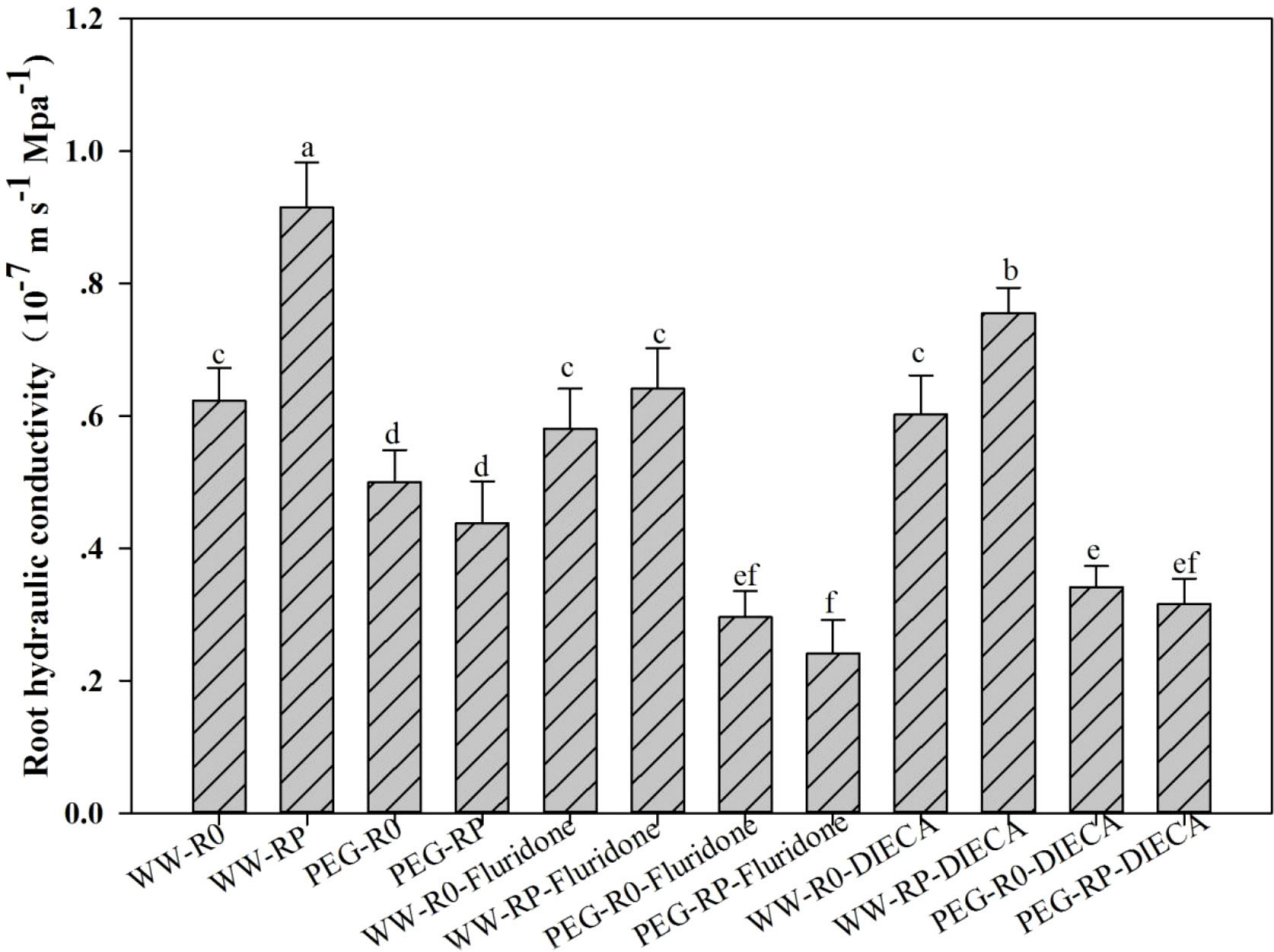
Effects of DIECA (100 mM; JA inhibitor) and fluridone (10 mM; ABA inhibitor) on root Lpr at 48 h. Data for three biological replicates were analysed by ANOVA. Different letters indicate statistically significant differences from each other (*P*< 0.05). FW, fresh weight.

The effects of the ABA inhibitor (fluridone) and the JA inhibitor (DIECA) on the post-pruning root Lpr were determined (**Figure 8**). Fluridone prevented an increase in Lpr after root pruning. Under well-watered conditions, the fluridone treatment reduced Lpr by 29.9% in RP. Under PEG stress, the fluridone treatment reduced Lpr by 45.0% in RP and 40.8% in R0. DIECA only partially inhibited an increase in Lpr after root pruning. DIECA treatment reduced Lpr by 17.5% in RP under well-watered conditions and by 31.6% in R0 and 27.8% in RP under PEG stress.

## 4 Discussion

Root pruning reduces soil water consumption by decreasing transpiration early in plant development. It conserves soil water, facilitates its transport to the shoots after flowering, promotes grain filling, and improves yield (Ma et al., 2008; Fang et al., 2010). In the present study, small root pruning significantly lowered maize root biomass and increased yield by 12.9% under well-watered conditions. Therefore, appropriate root pruning can improve maize yield (Chai et al., 2002). Root pruning increases photosynthetic activity and promotes grain filling (Ma et al., 2010). As also observed in our study that root pruning increased the photosynthetic rate in maize under well-watered conditions (**Figure S2**). For these reasons, plants with pruned roots exhibited a higher yield than those with unpruned roots. After root pruning, transpiration decreased; however, the yield of pruned plants was higher or similar to unpruned plants. Hence, plants with pruned roots exhibited a relatively higher WUE (**Table 2**). Thus, root pruning may improve both yield and WUE. Our findings also demonstrated that in arid and semi-arid regions, breeding drought resistance to increase yield and WUE should not be limited to cultivars with large root systems.

In normally growing plants, a dynamic balance exists between root water uptake and leaf water loss. A reasonable explanation is that root pruning may reduce the water supply from the root to the above-ground part, leading to a decline in leaf water potential. In this study, the leaf water potential of plants with pruned roots was not lower than that of plants with intact roots (**Figure 4**), which might be because residual roots may still transport sufficient water after pruning. Moreover, root pruning decreased transpiration rate (**Figure S2**). In our study we found that root pruning decreased the leaf water potential under PEG stress, which may be that root pruning may rapidly increase root sensitivity to PEG stress and simultaneous root pruning and PEG cause severe water deficit and upset the dynamic water balance.

A previous study reported that Lpr of the residual roots significantly increased within a few hours after 4/5 of the root system was excised (Vysotskaya et al., 2004). Plants with only a single root axis can absorb enough water to maintain normal transpiration possibly because of an increase in Lpr (Shane and McCully, 1999). In the present study, Lpr of the pruned plants was significantly higher than that of the unpruned plants at the jointing stage (**Figure 2**). We also observed the same results in the hydroponics experiments. The increase in Lpr in the single root axis may augment water availability which, in turn, recovers shoot growth (Vysotskaya et al., 2004). The transpiration rate significantly decreased whilst the water conductivity of the residual roots increased. The leaf water potential of plants with pruned roots was not significantly lower than that of plants with intact roots. Therefore, the plants formed a new water balance after root pruning. In our study the Lpr of pruned plants was not significantly (*P* > 0.05) different from that of the control at the anthesis and milk stages. Root pruning leads to a reduced R/S ratio during the jointing stage; however, there is a controlling mechanism balancing the growth of above- and below-ground plant parts (Vysotskaya et al., 2001). This mechanism enables plants to restore their R/S ratio after root pruning. In the present study, there was no significant difference in R/S ratio and Lpr between pruned plants and control at anthesis and milk stages, respectively. However, Lpr decreased after anthesis (**Figure 2**), which may be due to decreased root activity after anthesis (Wang et al., 2013).

Drought stress may reduce root water absorption capacity (Li et al., 2020). In our study, the complemented water was required to achieve the low moisture level (35–45% field water capacity), which may be alleviated drought stress. However, overall moisture was maintained at a low level; hence, the effects of alleviating drought stress may be relatively small. We determined that Lpr was significantly decreased under drought stress (**Figures 2**, **3**). This was due to difficulties associated with measuring the expression of aquaporin and hormone content involved in root water absorption in the pot experiment; therefore, we designed the hydroponic experiment to complement the soil study. In the pot experiment, root pruning increased Lpr under drought stress. PEG was chosen to simulate drought stress in hydroponic experiment. Winzor (2004) indicated that the theoretical or measured concentration-osmotic potential relations for PEG of different molecular weights can vary to some extent depending on the medium and the specific PEG used. In the hydroponic experiment, the same batch of PEG6000 was used to ensure the same molecular weight in our study. To prevent PEG from entering plant cells through the broken roots, we sealed the incision with paraffin immediately after root pruning and aerated it using an air pump in nutrient solution to reduce PEG damage. However, root pruning decreased Lpr under PEG stress (**Figure 3**), which differed to the pot experiment. Root pruning may rapidly increase root sensitivity to PEG stress. PEG treatment can instantaneously alter cell membrane permeability, damage plant cells, and rapidly induce water loss. When the root cap and hydraulic structure are not fully formed, osmoregulation and phytohormone levels are adjusted in an attempt to allow the plant to adapt to the stress. Simultaneous root pruning and PEG cause severe water deficit and upset the dynamic water balance. Hence, plant sensitivity to drought stress increases and Lpr quickly decreases. In the pot experiment, soil drought stress was gradual and did not cause rapid water loss from the plant cells. Hence, the plants re-established water homeostasis after root pruning. Under drought stress, Lpr was higher in the plants with pruned roots than in the control plants (**Figure 2**).

The water absorption capacity of crop root system depends on the absorption capacity of the unit root system. The increase in active absorption area of root system and root activity can significantly improve the water absorption capacity of the crop under early season drought or terminal drought (Wang and Shangguan, 2017; Figueroa-Bustos et al., 2019; Figueroa-Bustos et al., 2020). Plants with pruned roots exhibited higher root activity than those with intact roots; root activity was rapidly activated after pruning, and an increase in the activity of the remaining roots compensated for the removed part of the root system (**Figure 5**), as previously reported (Ma et al., 2013; Fanello et al., 2020).

AQP promotes root water uptake by improving water conduction through the symplast (Knipfer and Fricke, 2010; Knipfer et al., 2011). In this way, AQP promotes water conductivity in the entire root. PIPs play a key role in regulating Lpr (Abdelhakam et al., 2021). Here, *ZmPIP1:1*, *ZmPIP1:5, ZmPIP2:2* and *ZmPIP2:5* were highly upregulated and their expression levels peaked at 3 h after root pruning (**Figure 6**). Their regulatory patterns were similar to that of Lpr (**Figure 3**). Thus, residual root hydraulic conductivity may have increased in response to *ZmPIP*1:1, *ZmPIP*1:5, *ZmPIP*2:2, and *ZmPIP*2:5 upregulation after root pruning. Direct exposure of roots to water stress usually results in inhibition of aquaporin activity and water transport at the cell and whole organ levels (Maurel et al., 2015), which is also consistent with our results (**Figure 6**). During drought conditions, plant water potential declines, plant hydraulic resistance increases, and the extent of xylem vessel embolisation also increases in parallel with a decrease in aquaporin gene expression (Secchi et al., 2007). A significant reduction in accumulation of PIP transcript has been also observed in many plant species, such as Arabidopsis (Alexandersson et al., 2005), canola (Secchi et al., 2007), barley (Kurowska et al., 2019), and maize (Zhang et al., 2021; Quiroga et al., 2017).

Roots are among the primary agents in plants responsible for sensing and responding to environmental signals. Phytohormones such as ABA play a key role in regulating Lpr (Thompson et al., 2007; Tardieu et al., 2010; Ullah et al., 2018). ABA is an integral component of the mechanism improving plant adaption to water deficit (Schraut et al., 2005; Borel et al., 2010). The results of this experiment showed that the relative root ABA increased at 3 h after root pruning (**Figure 7A**). ABA may have been transported basipetally from the stem to the reduced root system and ABA biosynthesis and accumulation increase under drought stress (**Figure 7A**). ABA accelerates root water uptake and improves water transport within a plant (Rowe et al., 2016; Canales et al., 2021). The results of the experiment suggested that root ABA accumulation may induce aquaporin genes and increase Lpr after root pruning.

JA also plays a vital role in plant water uptake (Li et al., 2003). In the present study, root JA content was significantly higher under WW-RP than under WW-R0. Water deficit increases JA concentrations in several plant species (Chen et al., 2016). The root JA content gradually increased in response to PEG-induced drought stress (**Figure 7B**). Mutations in certain steps of JA biosynthesis and signalling pathways alter water deficit tolerance (Riemann et al., 2015). A previous study reported that JA can induce ABA synthesis (Adie et al., 2007) which, in turn, increases Lpr (Aroca, 2006; Mahdieh and Mostajeran, 2009). In the present study, the trends in root JA and ABA content were similar after root pruning (**Figure 7**) and may contribute to the observed root hydraulic regulation. JA might affect ABA biosynthesis and vice-versa (Fragoso et al., 2014). To verify whether Lpr improvement is related to increases in root ABA and JA content, we added DIECA (JA inhibitor) and fluridone (ABA inhibitor) to the nutrient solution, respectively. Root pruning increased Lpr in an ABA-independent manner. Fluridone abolished the impact of root pruning on Lpr whereas JA inhibitors only partially inhibited Lpr (**Figure 8**). Therefore, JA may indirectly regulate Lpr by increasing the ABA content and directly regulate Lpr in an ABA-independent manner.

Significant shifts in root growth, morphology, and physiology have frequently been observed after root pruning. Lateral root growth was greatly enhanced in *Platycladus orientalis* after root pruning (Feng et al., 2022). Compensatory increases in the specific root length, fine root vitality, and the ratio of fine roots to the total root mass have been reported in rice (Kawai et al., 2022), soybean (Fanello et al., 2020), and *Cunninghamia lanceolata* (Dong et al., 2016). Root pruning may promote lateral root growth and compensation growth (Kawai et al., 2022), thereby increasing the IAA and SA demand. Root IAA content increased approximately 10 times at 2 h after partial root resection (Vysotskaya et al., 2001). In our study, the root IAA and SA levels were significantly higher in the pruned roots than the control roots (WW-R0) between 3 h and 24 h after root pruning (**Figures 7B, D**). IAA regulates root growth, lateral root development, and flowering (Chandler, 2009). Both SA and IAA restored the R/S after root pruning. The observed increases in IAA and SA content after pruning may be explained by compensatory root growth and subsequent lateral root development. However, the IAA and SA content significantly decreased in response to PEG stress (**Figures 7B, D**). Both phytohormones are synthesised in the shoot apical meristem and leaves, and drought stress negatively affect these organs. Hence, there are balanced relationships among the IAA and SA content and plant growth under stress conditions.

## 5 Conclusions

The experimental results revealed that proper root pruning can improve maize yield and WUE. Plants with pruned roots did not have significantly lower leaf water potential than those with intact roots. Root pruning increased hydraulic conductivity in the residual roots by upregulating *ZmPIP*1:1*, ZmPIP*1:5*, ZmPIP*2:2 and *ZmPIP*2:5 and by modulating ABA and JA signalling.

## Data availability statement

The original contributions presented in the study are included in the article/**Supplementary Materials**, further inquiries can be directed to the corresponding author/s.

## Author contributions

SZ conceived and designed the experiment. Material preparation, data collection and analysis were performed by MY. The first draft of the manuscript was written by MY. CZ, HL, LZ, YR, YC and HC contributed to the writing and revision. All authors contributed to the article and approved the submitted version.

## References

[B1] Abdelhakam S. Rabei S. H. Nada R. M. Abogadallah G. M. (2021). The complementary role of root and leaf PIP1 and PIP2 aquaporins drives the anisohydric behavior in *Helinathus annuus* L. Environ. Exp. Bot. 182, 104314. doi: 10.1016/j.envexpbot.2020.104314

[B2] Adie B. A. T. Perez-Perez J. N. Perez-Perez M. M. Godoy M. Sanchez-Serrano J. J. Schmelz E. A. . (2007). ABA is an essential signal for plant resistance to pathogens affecting JA biosynthesis and the activation of defenses in arabidopsis. Plant Cell. 19, 1665–1681. doi: 10.1105/tpc.106.048041 17513501 PMC1913739

[B3] Alexandersson E. Fraysse L. Sjövall-Larsen S. Gustavsson S. Fellert M. Karlsson M. . (2005). Whole gene family expression and drought stress regulation of aquaporins. Plant Mol. Biol. 59 (3), 469–484. doi: 10.1007/s11103-005-0352-1 16235111

[B4] Aroca R. (2006). Exogenous catalase and ascorbate modify the effects of abscisic acid (ABA) on root hydraulic properties in *Phaseolus vulgaris* L. plants. J. Plant Growth Regul. 25, 10–17. doi: 10.1007/s00344-005-0075-1

[B5] Aroca R. Vernieri P. Irigoyen J. J. Sanchez-Diaz M. Tognoni F. Pardossi A. (2003). Involvement of abscisic acid in leaf and root of maize (*Zea mays* L.) in avoiding chilling-induced water stress. Plant Sci. 165, 671–679. doi: 10.1016/s0168-9452(03)00257-7

[B6] Aziz M. M. Palta J. A. Siddique K. H. M. Sadras V. O. (2017). Five decades of selection for yield reduced root length density and increased nitrogen uptake per unit root length in Australian wheat varieties. Plant Soil 413, 81–92. doi: 10.1007/s11104-016-3059-y

[B7] Barrero J. M. Talbot M. J. White R. G. Jacobsen J. V. Gubler F. (2009). Anatomical and transcriptomic studies of the coleorhiza reveal the importance of this tissue in regulating dormancy in barley. Plant Physiol. 150, 1006–10021. doi: 10.1104/pp.109.137901 19386806 PMC2689963

[B8] Borel C. Frey A. Marion-Poll A. Tardieu F. Simonneau T. (2010). Does engineering abscisic acid biosynthesis in *Nicotiana plumbaginifolia* modify stomatal response to drought? Plant Cell Environ. 24, 477–489. doi: 10.1046/j.1365-3040.2001.00698.x

[B9] Budiarto R. Poerwanto R. Santosa E. Efendi D. (2019). A review of root pruning to regulate citrus growth. J. Trop. Crop Sci. 6, 1–7. doi: 10.29244/jtcs.6.01.1-7

[B10] Canales F. J. Rispail N. Garcia-Tejera O. Arbona V. Perez-De-Luque A. Prats E. (2021). Drought resistance in oat involves ABA-mediated modulation of transpiration and root hydraulic conductivity. Environ. Exp. Bot. 182, 104333. doi: 10.1016/j.envexpbot.2020.104333

[B11] Chai S. W. Liu W. Z. Li Y. Y. (2002). Effect of root cutting on leaf photosynthesis rate and water use efficiency of maize. Chin. J. Appl. Ecol. 12, 1716–1718. doi: 10.1006/jfls.2001.0409 12682990

[B12] Chandler J. W. (2009). Auxin as compere in plant hormone crosstalk. Planta. 231, 1–12. doi: 10.1007/s00425-009-1036-x 19888599

[B13] Chen H. Y. Hsieh E. J. Cheng M. C. Chen C. Y. Hwang S. Y. Lin T. P. (2016). ORA47 (octadecanoid-responsive AP2/ERF-domain transcription factor 47 regulates jasmonic acid and abscisic acid biosynthesis and signaling through binding to a novel ciselement. New Phytol. 211, 599–613. doi: 10.1111/nph.13914 26974851

[B14] Corales M. Nguyen N. T. A. Abiko T. Mochizuki T. (2020). Mapping quantitative trait loci for water uptake of rice under aerobic conditions. Plant Prod. Sci. 23, 436–451. doi: 10.1080/1343943x.2020.1766361

[B15] Dong T. F. Duan B. L. Zhang S. Korpelainen H. N. Li C. Y. (2016). Growth biomass allocation and photosynthetic responses are related to intensity of root severance and soil moisture conditions in the plantation tree *Cunninghamia lanceolata* . Tree Physiol. 36, 807–817. doi: 10.1093/treephys/tpw025 27122365

[B16] Du M. M. Zhao J. H. Tzeng D. T. W. Liu Y. Y. Deng L. Yang T. X. . (2017). MYC2 orchestrates a hierarchical transcriptional cascade that regulates jasmonate-mediated plant immunity in tomato. Plant Cell. 29, 1883–1906. doi: 10.1105/tpc.16.00953 28733419 PMC5590496

[B17] Ehdaie B. Merhaut D. J. Ahmadian S. Hoops A. C. Khuong T. Layne A. P. . (2010). Root system size influences water-nutrient uptake and nitrate leaching potential in wheat. J. Agron. Crop Sci. 196, 455–466. doi: 10.1111/j.1439-037X.2010.00433.x

[B18] Fanello D. D. Kelly S. J. Bartoli C. G. Cano M. G. Alonso S. M. Guiamet J. J. (2020). Plasticity of root growth and respiratory activity: Root responses to above-ground senescence, fruit removal or partial root pruning in soybean. Plant Sci., 290, 110296. doi: 10.1016/j.plantsci.2019.110296 31779891

[B19] Fang Y. Liang L. Liu S. Xu B. C. Siddique K. H. M. Palta J. A. . (2021). Wheat cultivars with small root length density in the topsoil increased post-anthesis water use and grain yield in the semi-arid region on the loess plateau. Eur. J. Agron. 124, 126243. doi: 10.1016/j.eja.2021.126243

[B20] Fang Y. Xu B. C. Turner N. C. Li F. M. (2010). Does root pruning increase yield and water-use efficiency of winter wheat? Crop Pasture Sci. 61, 899–910. doi: 10.1071/CP10125

[B21] Feng Z. Kong D. Kong Y. Zhang B. Yang X. (2022). Coordination of root growth with root morphology, physiology and defense functions in response to root pruning in *Platycladus orientalis* . J. Adv. Res. 36, 187–199. doi: 10.1016/j.jare.2021.07.005 35127173 PMC8799911

[B22] Figueroa-Bustos V. Palta J. A. Chen Y. Siddique K. H. M. (2019). Early season drought largely reduces grain yield in wheat cultivars with smaller root systems. Plants. 8, 305. doi: 10.3390/plants8090305 31461902 PMC6783945

[B23] Figueroa-Bustos V. Palta J. A. Chen Y. Stefanova K. Siddique K. H. M. (2020). Wheat cultivars with contrasting root system size responded differently to terminal drought. Front. Plant Sci. 11, 1285. doi: 10.3389/fpls.2020.01285 32973844 PMC7466772

[B24] Fragoso V. Rothe E. Baldwin I. T. Kim S. G. (2014). Root jasmonic acid synthesis and perception regulate folivore-induced shoot metabolites and increase *Nicotiana attenuata* resistance. New Phytol. 202, 1335–1345. doi: 10.1111/nph.12747 24580101 PMC5156298

[B25] Haswell E. S. Verslues P. E. (2015). The ongoing search for the molecular basis of plant osmosensing. J. Gen. Physiol. 145, 389–394. doi: 10.1085/jgp.201411295 25870206 PMC4411250

[B26] Hellal F. El-Shabrawi H. Abd El-Hady M. Khatab I. El-Sayed S. Abdelly C. (2018). Influence of PEG induced drought stress on molecular and biochemical constituents and seedling growth of egyptian barley cultivars. J. Gen. Eng. Biotechnol. Adv. 16, 203–212. doi: 10.1016/j.jgeb.2017.10.009 PMC6296644 30647723

[B27] Hirte J. Leifeld J. Abiven S. Mayer J. (2018). Maize and wheat root biomass, vertical distribution, and size class as affected by fertilization intensity in two long-term field trials. Field Crops Res. 216, 197–208. doi: 10.1016/j.fcr.2017.11.023

[B28] Hu C. Sadras V. O. Lu G. Zhang R. Yang X. Zhang S. (2019). Root pruning enhances wheat yield, harvest index and water-use efficiency in semiarid area. Field Crops Res. 230, 62–71. doi: 10.1016/j.fcr.2018.10.013

[B29] Kawai T. Chen Y. Takahashi H. Inukai Y. Siddique K. H. M. (2022). Rice genotypes express compensatory root growth with altered root distributions in response to root cutting. Front. Recent Dev. Plant Sci. 13, 830577. doi: 10.3389/fpls.2022.830577 PMC8919052 35295630

[B30] Knipfer T. Besse M. Verdeil J. L. Fricke W. (2011). Aquaporin-facilitated water uptake in barley (*Hordeum vulgare* L.) roots. J. Exp. Bot. 62, 4115–4126. doi: 10.1093/jxb/err075 21441404 PMC3153672

[B31] Knipfer T. Fricke W. (2010). Root pressure and a solute reflection coefficient close to unity exclude a purely apoplastic pathway of radial water transport in barley (*Hordeum vulgare*). New Phytol. 187, 159–170. doi: 10.1111/j.1469-8137.2010.03240.x 20412443

[B32] Kurowska M. M. Wiecha K. Gajek K. Szarejko I. (2019). Drought stress and re-watering affect the abundance of TIP aquaporin transcripts in barley. PLoS One 14 (12), e0226423. doi: 10.1371/journal.pone.0226423 PMC6917287 31846477

[B33] Lee S. H. Chung G. C. Jang J. Y. Ahn S. J. Zwiazek J. J. (2012). Overexpression of *PIP2:5* aquaporin alleviates effects of low root temperature on cell hydraulic conductivity and growth in arabidopsis. Plant Physiol. 159, 479–488. doi: 10.1104/pp.112.194506 22434042 PMC3375980

[B34] Li Y. Li S. He X. Jiang W. Zhang D. Li B. . (2020). CO2 enrichment enhanced drought resistance by regulating growth, hydraulic conductivity and phytohormone contents in the root of cucumber seedlings. Plant Physiol. Biochem. 152, 62–71. doi: 10.1016/j.plaphy.2020.04.037 32388421

[B35] Li C. Y. Liu G. H. Xu C. C. Lee G. I. Bauer P. Ling H. Q. . (2003). The tomato suppressor of prosystemin-mediated responses 2 gene encodes a fatty acid desaturase required for the biosynthesis of jasmonic acid and the production of a systemic wound signal for defense gene expression. Plant Cell. 15, 1646–1661. doi: 10.1105/tpc.012237 12837953 PMC165407

[B36] Liu Z. Liu B. H. Li Y. C. (2007). Effects of root-cutting on photosynthesis and growth of winter wheat at late stage of rising stage. Acta Agric. Boreali-Sinica. 22, 189–190. doi: 10.7668/hbnxb.2007.05.045

[B37] Luo Z. Kong X. Zhang Y. (2019). Leaf-derived jasmonate mediates water uptake from hydrated cotton roots under partial root-zone irrigation. Plant Physiol. 180, 1660–1676. doi: 10.1104/pp.19.00315 31079035 PMC6752905

[B38] Mahdieh M. Mostajeran A. (2009). Abscisic acid regulates root hydraulic conductance *via* aquaporin expression modulation in *Nicotiana tabacum* . J. Plant Physiol. 166, 1993–2003. doi: 10.1016/j.jplph.2009.06.001 19576659

[B39] Ma S. C. Li F. M. Xu B. C. Huang Z. B. (2008). Effects of root pruning on the growth and water use efficiency of winter wheat. Plant Growth Regul. 57, 233–241. doi: 10.1007/s10725-008-9340-1

[B40] Ma S. C. Li F. M. Xu B. C. Huang Z. B. (2010). Effect of lowering the root/shoot ratio by pruning roots on water use efficiency and grain yield of winter wheat. Field Crops Res. 115, 158–164. doi: 10.1016/j.fcr.2009.10.017

[B41] Ma S. C. Li F. M. Yang S. J. Li C. X. Xu B. C. Zhang X. C. (2013). Effects of root pruning on non-hydraulic root-sourced signal, drought tolerance and water use efficiency of winter wheat. J. Integr. Agric. 12, 989–998. doi: 10.1016/s2095-3119(13)60476-1

[B42] Martre P. Morillon R. Barrieu F. North G. B. Nobel P. S. Chrispeels M. J. (2002). Plasma membrane aquaporins play a significant role during recovery from water deficit. Plant Physiol. 130, 2101–2110. doi: 10.1104/pp.009019 12481094 PMC166722

[B43] Maurel C. Boursiac Y. Luu D. T. Santoni V. Shahzad Z. Verdoucq L. (2015). Aquaporins in plants. Physiol. Rev. 95 (4), 1321–1358. doi: 10.1152/physrev.00008.2015 26336033

[B44] Meister R. Rajani M. S. Ruzicka D. Schachtman D. P. (2014). Challenges of modifying root traits in crops for agriculture. Trends Plant Sci. 19, 779–788. doi: 10.1016/j.tplants.2014.08.005 25239776

[B45] Meng D. L. Fricke W. (2017). Changes in root hydraulic conductivity facilitate the overall hydraulic response of rice (*Oryza sativa* L.) cultivars to salt and osmotic stress. Plant Physiol. Biochem. 113, 64–77. doi: 10.1016/j.plaphy.2017.02.001 28189051

[B46] Olaetxea M. Mora V. Bacaicoa E. (2015). Abscisic acid regulation of root hydraulic conductivity and aquaporin gene expression is crucial to the plant shoot growth enhancement caused by rhizosphere humic acids. Plant Physiol. 169, 2587–2596. doi: 10.1104/pp.15.00596 26450705 PMC4677878

[B47] Palta J. A. Chen X. Milroy S. P. Rebetzke G. J. Dreccer M. F. Watt M. (2011). Large Root systems: Are they useful in adapting wheat to dry environments? Funct. Plant Biol. 38, 347–354. doi: 10.1071/fp11031 32480891

[B48] Passioura J. B. (1983). Roots and drought resistance. Agric. Water Manage. 7, 265–280. doi: 10.1016/0378-3774(83)90089-6

[B49] Perrone I. Gambino G. Chitarra W. (2012). The grapevine root-specific aquaporin *VvPIP2; 4N* controls root hydraulic conductance and leaf gas exchange under well-watered conditions but not under water stress. Plant Physiol. 160, 965–977. doi: 10.1104/pp.112.203455 22923680 PMC3461569

[B50] Qi T. C. Huang H. Song S. S. Xie D. X. (2015). Regulation of jasmonate-mediated stamen development and seed production by a *bHLH-MYB* complex in *Arabidopsis* . Plant Cell. 27, 1620–1633. doi: 10.1105/tpc.15.00116 26002869 PMC4498206

[B51] Quiroga G. Erice G. Aroca R. Chaumont F. Ruiz-Lozano J. M. (2017). Enhanced drought stress tolerance by the arbuscular mycorrhizal symbiosis in a drought-sensitive maize cultivar is related to a broader and differential regulation of host plant aquaporins than in a drought-tolerant cultivar. Front. Plant Sci. 8, 1056. doi: 10.3389/fpls.2017.01056 PMC5474487 28674550

[B52] Riemann M. Dhakarey R. Hazman M. Miro B. Kohli A. Nick P. (2015). Exploring jasmonates in the hormonal network of drought and salinity responses. Front. Plant Sci. 6, 1077. doi: 10.3389/fpls.2015.01077 PMC4665137 26648959

[B53] Rowe J. H. Topping J. F. Liu J. L. Lindsey K. (2016). Abscisic acid regulates root growth under osmotic stress conditions *via* an interacting hormonal network with cytokinin, ethylene and auxin. New Phytol. 211, 225–239. doi: 10.1111/nph.13882 26889752 PMC4982081

[B54] Sánchez-Romera B. Calvo-Polanco M. N. Ruiz-Lozano J. M. Zamarreno M. A. Arbona V. García-Mina J. M. . (2018). Involvement of the *def-1* mutation in the response of tomato plants to arbuscular mycorrhizal symbiosis under well-watered and drought conditions. Plant Cell Physiol. 59, 248–261. doi: 10.1093/pcp/pcx178 29165704

[B55] Schraut D. Heilmeier H. Hartung W. (2005). Radial transport of water and abscisic acid (ABA) in roots of *Zea* mays under conditions of nutrient deficiency. J. Exp. Bot. 56, 879–886. doi: 10.1093/jxb/eri080 15699064

[B56] Secchi F. Lovisolo C. Schubert A. (2007). Expression of *OePIP2; 1* aquaporin gene and water relations of *Olea europaea* twigs during drought stress and recovery. Ann. Appl. Biol. 150 (2), 163–167. doi: 10.1111/j.1744-7348.2007.00118.x

[B57] Shane M. W. McCully M. E. (1999). Root xylem embolisms: implications for water flow to the shoot in single-rooted maize plants. Aust. J. Plant Physiol. 26, 107–114. doi: 10.1071/pp98060

[B58] Song L. Li F. Fan X. (2009). Soil water availability and plant competition affect the yield of spring wheat. Eur. J. Agron. 31, 51–60. doi: 10.1016/j.eja.2009.03.003

[B59] Sreeman S. M. Vijayaraghavareddy P. Sreevathsa R. Rajendrareddy S. Arakesh S. Bharti P. . (2018). Introgression of physiological traits for a comprehensive improvement of drought adaptation in crop plants. Front. Chem. 6, 382. doi: 10.3389/fchem.2018.00092 PMC6118222 30186834

[B60] Tardieu F. Parent B. Simonneau T. (2010). Control of leaf growth by abscisic acid: Hydraulic or non-hydraulic processes? Plant Cell Environ. 33, 636–647. doi: 10.1111/j.1365-3040.2009.02091.x 20002334

[B61] Thompson A. J. Andrews J. Mulholland B. J. (2007). Overproduction of abscisic acid in tomato increases transpiration efficiency and root hydraulic conductivity and influences leaf expansion. Plant Physiol. 143, 1905–1917. doi: 10.1104/pp.106.093559 17277097 PMC1851808

[B62] Ullah A. Manghwar H. Shaban M. Khan A. H. Akbar A. Ali U. . (2018). Phytohormones enhanced drought tolerance in plants: A coping strategy. Environ. Sci. pollut. Res. Int. 25, 33103–33118. doi: 10.1007/s11356-018-3364-5 30284160

[B63] Vysotskaya L. B. Arkhipova T. N. Timergalina L. N. Dedov A. V. Veselov S. Y. Kudoyarova G. R. (2004). Effect of partial root excision on transpiration, root hydraulic conductance and leaf growth in wheat seedlings. Plant Physiol. Biochem. 42, 251–257. doi: 10.1023/a:1010700617829 15051049

[B64] Vysotskaya L. B. Timergalina L. N. Simonyan M. V. Veselov S. Y. Kudoyarova G. R. (2001). Growth rate, IAA and cytokinin content of wheat seedling after root pruning. Plant Growth Regul. 33, 51–57. doi: 10.1023/A:1010700617829

[B65] Wang Z. Y. Lv J. Y. Li F. M. Xu B. C. (2007). Effect of root excision on competitive ability and yield of winter wheat. Plant Ecol. 31, 300–304. doi: 10.17521/cjpe.2007.0034

[B66] Wang X. B. Shangguan Z. P. (2017). Effect of nitrogen on root vigor and growth in different genotypes of wheat under drought stress. J. Triticeae. Crops. 37, 820–827. doi: 10.7606/j.issn.1009-1041,2017.06.014

[B67] Wang X. L. Zhang S. Q. Shan L. (2013). Effects of cultivars intercropping on maize water balance under different planting densities. Chin. J. Eco-Agric. 21 (2), 171–178. doi: 10.3724/SP.J.1011.2013.00171

[B68] Winzor D. J. (2004). Reappraisal of disparities between osmolality estimates by freezing point depression and vapor pressure deficit methods. Biophys. Chem. 107, 317–323. doi: 10.1016/j.bpc.2003.11.010 14967246

[B69] Xu Z. H. Liang M. L. Lu D. X. (2016). Effect of cutting roots vertically at a place with different horizontal distance from plant on yield and grain storage capacity of summer maize. Acta Agron. Sin. 42, 1805–1816. doi: 10.3724/SP.J.1006.2016.01805

[B70] Yao C. Zhang F. Sun X. Shang D. He F. Li X. . (2019). Effects of s-abscisic acid (S-ABA) on seed germination, seedling growth, and asr1 gene expression under drought stress in maize. J. Plant Growth Regul. 38, 1300–1313. doi: 10.1007/s00344-019-09934-9

[B71] Zhang J. H. Jia W. S. Yang J. C. Ismail A. M. (2006). Role of ABA in integrating plant responses to drought and salt stresses. Field Crops Res. 97, 111–119. doi: 10.1016/j.fcr.2005.08.018

[B72] Zhang L. Yan M. F. Ren Y. Y. Chen Y. L. Zhang S. Q. (2021). Zinc regulates the hydraulic response of maize root under water stress conditions. Plant Physiol. Biochem. 159, 123–134. doi: 10.1016/j.plaphy.2020.12.014 33360236

