# Supplementary material for: Root pruning improves maize water-use efficiency by root water absorption
Source: Front Plant Sci. 2023 Jan 4;13:1023088. doi: 10.3389/fpls.2022.1023088 (PMC9845614; doi:10.3389/fpls.2022.1023088)
Supplement: Supplementary file 1 [file DataSheet_1.docx]

**Supplementary material**

**Table S1**. Primers used in quantitative real-time PCR

| Gene | Primer sequence | |
| --- | --- | --- |
|  | Forward primer | Reverse primer |
| ZmPIPl;1 | CCCCTACTATGTTACGTGGAGTTC | GCGGCATATTACACAATTGGTA |
| ZmPIPl;2 | CTCATTTTATGCGTTGGGATGT | ACTGAAACCAAGAAAACCCTGA |
| ZmPIPl;3 | GGTTCCCGTATCCTTTTATGC | AATCCAGCTGATAGATAAACCCAC |
| ZmPIPl;4 | GCCATCTACCACCAGGTGAT | GGGCAGACAATACATTCCCC |
| ZmPIPl;5 | CACGTGGTCATCATCAGGG | CGTATGCTGCATGGTTGCT |
| ZmPIP2;l | CGGGTCGCCTTTTTTTTG | CCCTTGAGAGTCACGACATGA |
| ZmPIP2;2 | GGCCTTCTACCACCAGTACATC | GGCCTTTCTTTAGCTCTGCTC |
| ZmPIP2;4 | TACCGGAGCAACGCCTAAG | GAAAACAGCAGCGAGCGA |
| ZmPIP2;5 | TGTCGTCGTTGGTTGCCT | CACAACAATCACACTAGCTTGGAA |
| ZmPIP2;6 | TTTAAGGTGAACGGAGAAGGAGA | GAAAGCTACTGCTGCTGTGGAT |
| GADPH | AGCAGGTCGAGCATCTTCG | CTGTAGCCCCACTCGTTGTC |


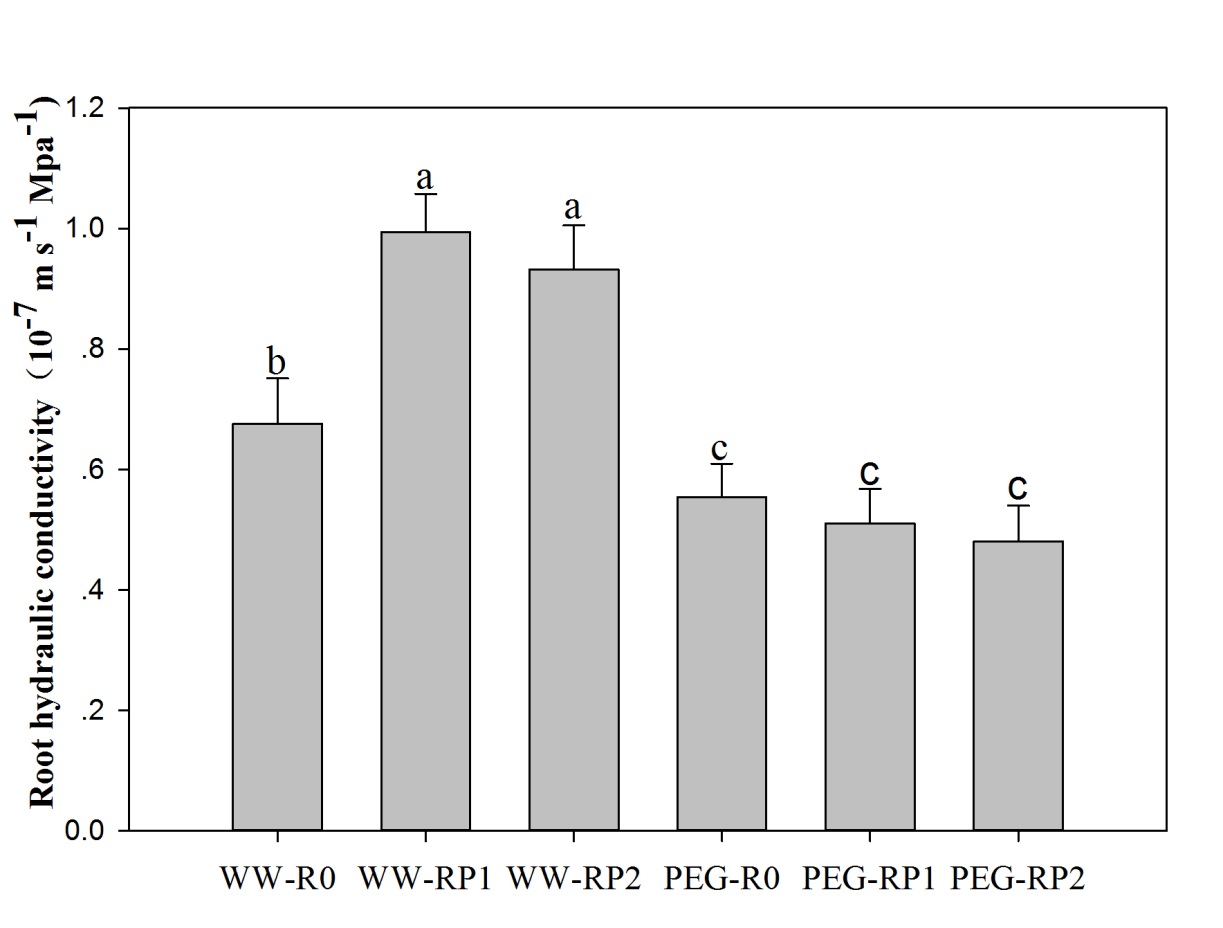


**Fig. S1. Effects of root pruning on Lpr in the preliminary experiments under hydroponic.** WW-R0 is no root pruning under well-watered conditions; WW-RP1 is removal of 1/5 of the root system under well-watered conditions; WW-RP2 is removal of 1/3 of the root system under well-watered conditions; PEG-R0 is no root pruning under PEG stress; PEG-RP1 is PEG stress plus removal of 1/5 of the root system; PEG-RP2 is PEG stress plus removal of 1/3 of the root system at 48 h after treatment. Values are means ± SD (n=6). Different letters indicate significant differences among treatments (p < 0.05) based on Duncan’s test.


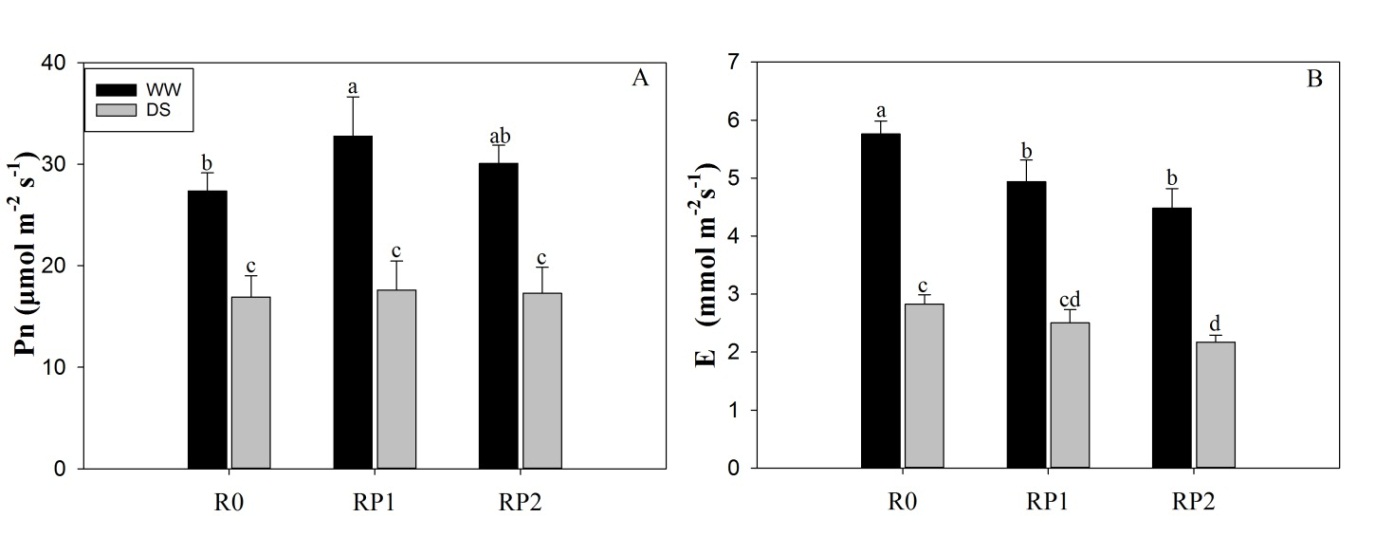


**Fig. S2. Effects of leaf gas exchange characteristics in the pot experiment.** Photosynthetic rate (Pn) (A) and transpiration rate (E) (B), **W**W-R0 is no root pruning under well-watered conditions; WW-RP1 is small root pruning under well-watered conditions; WW-RP2 is large root pruning under well-watered conditions; DS-R0 is no root pruning under drought stress; DS-RP1 is small root pruning under drought stress; DS-RP2 is large root pruning under drought stress. Samples were measured at anthesis. Values are means ± SD (n=6). Different letters indicate significant differences among treatments (p < 0.05) based on Duncan’s test.
